# Diagnostic Challenges in Nephrotic Syndrome Presenting With Venous Thromboembolism

**DOI:** 10.7759/cureus.71173

**Published:** 2024-10-09

**Authors:** Abhinav R Thummala, Tyler Morad, Christopher Lees, Emily E Cantor

**Affiliations:** 1 Internal Medicine, University of California Los Angeles David Geffen School of Medicine, Los Angeles, USA; 2 Hospital Medicine, Veterans Affairs (VA) Greater Los Angeles Health Care, Los Angeles, USA

**Keywords:** adult nephrotic syndrome, deep vein thrombosis (dvt), hypercoagulability syndromes, kidney biopsy, minimal change disease (mcd), pulmonary embolism (pe)

## Abstract

Nephrotic syndrome (NS) has a well-established relationship with hypercoagulability and, while rare, is known to present with unprovoked venous thromboembolism (VTE). Here, we present a case of seemingly unprovoked deep vein thrombosis and pulmonary embolism as presenting features of NS. We explore the challenge of timing diagnostic renal biopsy with necessary therapeutic anticoagulation, particularly in patients who present with unstable or extensive VTE. We also examine relevant factors in selecting an anticoagulant and discuss emerging treatment modalities in NS. This case underscores the complexity of medical decision-making in NS presenting with VTE and highlights the importance of multi-disciplinary consideration of patient-specific risks and benefits.

## Introduction

Nephrotic syndrome (NS) classically presents with edema and fatigue, though patients are also at increased risk for associated infection, hypercoagulability, and hyperlipidemia. While NS is initially suspected with urine and serologic testing, renal biopsy remains the standard to confirm pathology and guide disease-specific therapy. Despite recent procedural advances and the use of ultrasonographic guidance, renal biopsy remains a high-risk procedure, with a post-procedure hemorrhage rate of 1.2% [1]. In patients presenting with venous thromboembolism (VTE), the need for anticoagulation may preclude renal biopsy, thereby delaying diagnosis and treatment. We present a case of NS-associated VTE that highlights the challenges of timing a renal biopsy, selection of anticoagulation, and the importance of a multidisciplinary framework in evaluation and treatment.

## Case presentation

**Figure 1:**
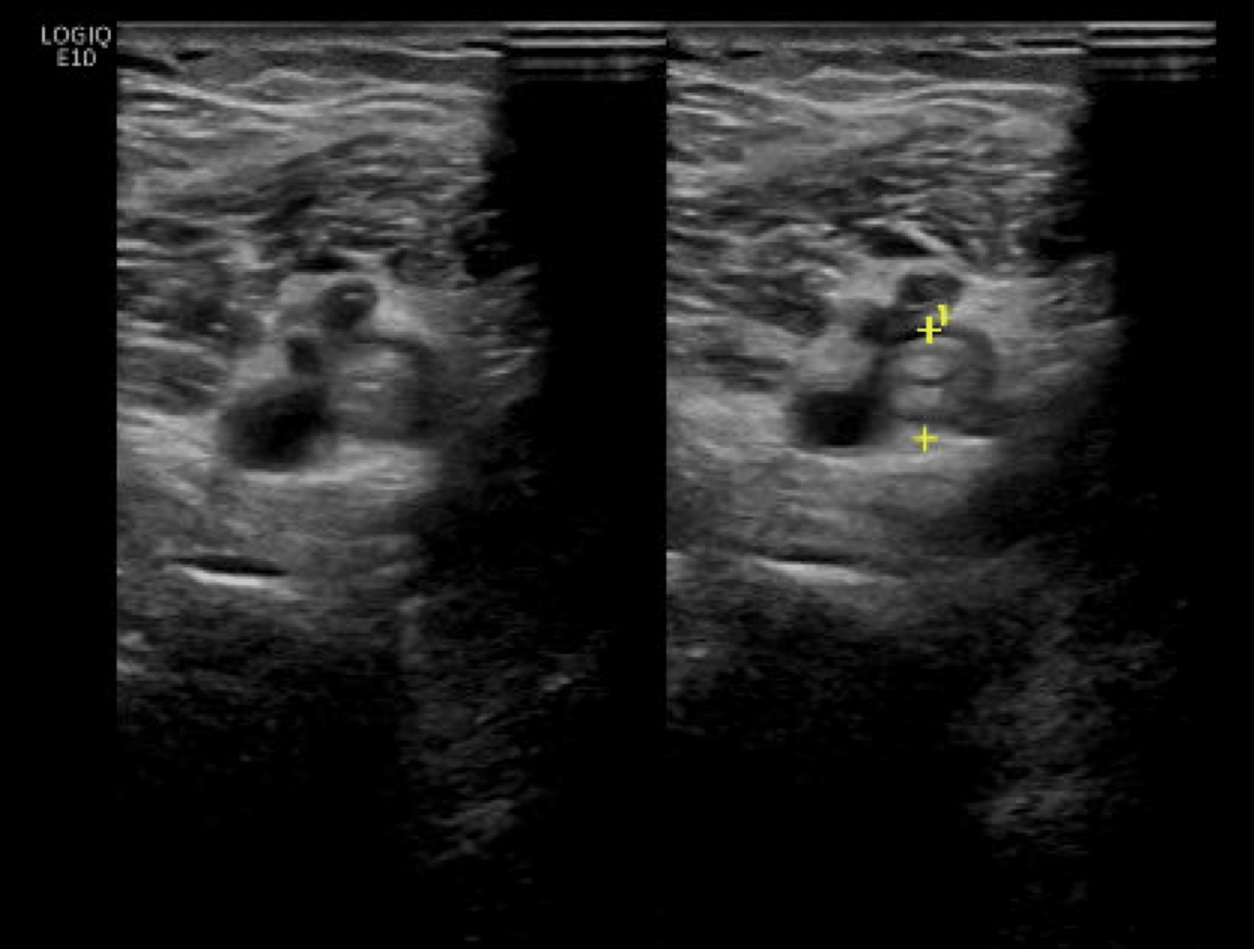
Deep vein thrombosis of the left popliteal vein (demarcated by yellow markers)

A 48-year-old man with a past medical history notable for alcohol use disorder and a 25-pack-year smoking history initially presented to the emergency department with three weeks of slowly progressive bilateral lower extremity edema and acute-onset left leg pain. He had no other known illnesses prior to the onset of symptoms, and he was not taking any medications prior to presentation. The patient’s leg swelling initially improved with the application of compression stockings until the day prior to presentation, at which time he noted sharp left leg pain associated with rapid progression of left lower extremity edema. Vitals on initial presentation included a temperature of 36.3 Celsius, a heart rate of 105, a blood pressure of 144/93, a respiratory rate of 18, and an oxygen saturation of 98% on room air. On exam, the left lower extremity was greater in circumference and more edematous compared to the right with edema extension to the knee. A Doppler ultrasound demonstrated deep vein thrombosis of the left popliteal and proximal femoral veins (Figure 1). Initial laboratory studies, including complete blood count, basic metabolic panel, hepatic transaminases, and brain natriuretic peptide, were interpreted as unrevealing. Notably, albumin was found to be 1.8 g/dL. The patient lacked historical features concerning provoked VTE and was discharged from the emergency department with therapeutic apixaban to treat deep vein thrombosis superimposed on presumed venous stasis.

**Figure 2:**
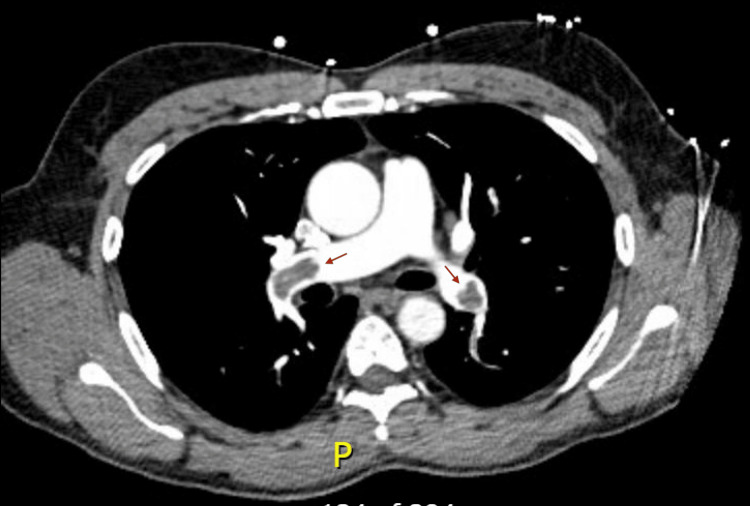
Representative image of the patient’s pulmonary emboli (marked with arrows) as seen on CT angiogram. The full study demonstrated extensive PE in the bilateral distal main, lobar, segmental, and subsegmental arteries

The patient was re-presented to the emergency department three days later with worsening left leg pain and progressive edema despite adherence to apixaban. These symptoms were also associated with new dyspnea on mild exertion, particularly with lifting heavy objects. The patient denied associated symptoms of chest pain, hemoptysis, dizziness, and palpitations. Vitals included a temperature of 97.6 Fahrenheit, a heart rate of 101 beats per minute, a blood pressure of 152/98, a respiratory rate of 18, and an oxygen saturation of 100% on room air. The physical exam re-demonstrated prior findings, though now with progressive left greater than right pitting edema to the thigh. The patient underwent an urgent CT angiogram of the chest (Figure 2), which demonstrated extensive pulmonary emboli in the bilateral distal main, lobar, segmental, and subsegmental pulmonary arteries without evidence of right heart strain. The transthoracic echocardiogram demonstrated normal right ventricular function; troponin was <3 ng/L, and BNP was 15 pg/dL. The patient was started on a therapeutic heparin infusion and admitted for further evaluation.

In light of the patient's previously identified hypoalbuminemia, urinalysis was performed on admission and demonstrated 3+ proteinuria. The urine total protein/creatinine ratio was 5.04, and subsequent 24-hour urine protein collection confirmed nephrotic range proteinuria at 5.2 g/day. Creatinine was 0.66 mg/dL, and serum albumin was 1.5 g/dL. Pending a definitive pathologic diagnosis, a broad workup for conditions associated with NS was performed. This serologic evaluation included testing for membranous nephropathy, systemic lupus erythematosus, large and small-vessel vasculitis, human immunodeficiency virus, hepatitis, and amyloidosis, all of which were negative. Nephrology was consulted and recommended a renal biopsy to differentiate between minimal change disease (MCD), focal segmental glomerulosclerosis, and membranous nephropathy, along with possible secondary causes of nephrosis. As therapeutic anticoagulation would need to be held for 48-72 hours prior to the procedure, pulmonology was consulted in light of the patient’s extensive clot burden. After considering the extent of the patient’s pulmonary emboli and the potential for rapid clinical worsening with premature discontinuation of anticoagulation, pulmonology recommended continuing uninterrupted anticoagulation for three months. A multidisciplinary discussion was arranged between the primary medicine team, nephrology, pulmonology, and interventional radiology to discuss the timing and safety of renal biopsy. Ultimately, the decision was made to continue three months of uninterrupted apixaban with outpatient renal biopsy thereafter. Empiric initiation of steroids was considered but ultimately deferred to maximize diagnostic yield with renal biopsy. Prior to discharge, an ACE inhibitor was empirically initiated to limit the extent of proteinuria, and outpatient monitoring of both blood pressure and proteinuria was planned with nephrology and pulmonology.

In the outpatient setting, the patient's urine total protein/creatinine ratio initially progressed to 10.9 before improving to 5.96 after continued ACE-inhibitor therapy. After three months of therapeutic anticoagulation, the patient tolerated the renal biopsy well without complication. Pathology showed diffuse foot process effacement with basement membrane thickening consistent with MCD and focal areas of sclerosis attributed to tobacco use. He was counseled on smoking cessation and initiated on tacrolimus-based therapy with daily prednisone. Now in remission, he continues to tolerate anticoagulation without episodes of bleeding or recurrent PE while on a slow tacrolimus and prednisone taper.

## Discussion

Our case highlights the importance of evaluating for underlying etiologies in a seemingly unprovoked case of VTE. After the diagnosis of NS is made via non-invasive studies, treatment decisions are heavily informed by the definitive pathologic diagnosis. The optimal timing of renal biopsy in patients with NS and extensive thromboembolic burden remains unknown. While there is no clear guidance on the safe timing of renal biopsy in cases of suspected NS with extensive VTE, the decision should be individualized and based on specific patient risk factors.

Although the definitive pathophysiology of hypercoagulability in NS remains unclear, many theories have been proposed, including loss of anticoagulants, enhanced platelet-vessel wall interaction, and increased fibrinogen conversion and fibrin deposition [2]. Hypercoagulability in NS was first described with renal vein thrombosis, though patients are also at higher risk for arterial and venous thrombosis at other sites [3]. Patients with new diagnoses of NS are often at the highest risk early in their disease course, with most thromboembolic events in adults occurring within six months of diagnosis [4]. The best predictors of VTE in NS include histologic diagnosis (membranous nephropathy carries the highest risk), degree of proteinuria, and serum albumin; as these urinary and serologic markers improve with treatment, the risk of VTE also decreases [5]. The rate of VTE in NS is thought to be higher in adults compared to children, though the exact incidence is difficult to estimate due to heterogeneity in the literature [6].

Treatment of NS-associated VTE has historically centered around warfarin as the anticoagulant of choice, though this paradigm is currently under active investigation. The efficacy of direct oral anticoagulants (DOACs) for therapeutic anticoagulation has not been adequately studied in this population, and most evidence supporting their use comes from case reports and small cohort studies [7-8]. A few studies have examined the role of DOACs for VTE prophylaxis in NS [9-11]. In one study, Kelddal et al. reported no VTE or major bleeding events for patients with NS and hypoalbuminemia treated with DOACs for VTE prophylaxis [7]. In spite of this growing evidence base, randomized control trials are necessary to further elucidate safety, efficacy, and bleeding rates for DOACs and warfarin for NS-associated VTE.

In this case, apixaban was continued given its aforementioned efficacy for VTE prophylaxis in NS, favorable side effect profile, and predictable therapeutic effect without the need for laboratory monitoring. It was determined that treatment failure was unlikely with the initial three days of apixaban, as it was presumed that his PE was probably present at the time of the initial presentation with DVT. In patients with true treatment failure on either warfarin or DOAC, most case studies support switching to an alternate class of anticoagulant [12-16]. Similarly, in cases of breakthrough VTE despite prophylactic anticoagulation, the anticoagulant is often transitioned to an alternate class [10].

While renal biopsy is typically a safe procedure, recent studies have re-demonstrated known bleeding risks. Microscopic hematuria is present in virtually all cases, and major bleeding complications may occur in up to 1.2% of percutaneous renal biopsies [1,17]. An earlier renal biopsy was considered while the patient was admitted on heparin, though it was ultimately deferred due to extensive pulmonary embolic burden as well as scheduling and staffing limitations. We propose that for suspected NS with significant thromboembolic burden, management of VTE should be prioritized to allow for clot stabilization with assessment of patient-specific variables to further guide therapeutic decision-making.

Minimal change disease is the most common cause of NS in children, though it is less commonly diagnosed in adults [18]. The treatment of MCD has historically been glucocorticoid monotherapy, with high rates of remission in pediatric trials [19]. While some studies demonstrate comparable long-term outcomes in adults and children after steroid therapy, other evidence suggests lower rates of early remission following glucocorticoid treatment in adults [20-21]. Due to these considerations and the frequency of drug-related adverse effects, glucocorticoid-sparing regimens may be employed. Previously described regimens include calcineurin inhibitors (tacrolimus) in conjunction with low-dose steroids [22-23].

Based on this emerging body of evidence, the patient was started on tacrolimus-based therapy with low-dose prednisone. Chin et al. reported that tacrolimus with low-dose prednisone was non-inferior to high-dose steroids in achieving remission for adult-onset minimal change disease, with the tacrolimus group demonstrating significantly lower relapse rates [22]. While calcineurin inhibitors have a unique side effect profile that must be monitored, this approach carries the advantage of avoiding the common side effects seen with high-dose, long-term corticosteroid therapy. In considering different therapeutic modalities following biopsy, shared decision-making with a discussion of short- and long-term risks and benefits best serves the patient.

## Conclusions

When diagnosing and treating a patient with VTE in the context of newly diagnosed NS, providers must consider multiple unique factors: selecting an appropriate anticoagulant, determining optimal timing to minimize the bleeding risk of renal biopsy, and ultimately identifying and treating the underlying etiology. At each complex decision point in this case, diagnostic and therapeutic efficacy depended on clear communication with the patient and multidisciplinary specialists, ultimately allowing for a result that best balanced the risks and benefits of his nephrotic-syndrome-specific factors. This case highlights the importance of patient-specific variables in complex medical decision-making and reinforces the need for ongoing research on anticoagulants in NS to better establish safety and efficacy.
